# Involvement of Elf3 on Smad3 activation-dependent injuries in podocytes and excretion of urinary exosome in diabetic nephropathy

**DOI:** 10.1371/journal.pone.0216788

**Published:** 2019-05-31

**Authors:** Akiko Sakurai, Hiroyuki Ono, Arisa Ochi, Motokazu Matsuura, Sakiya Yoshimoto, Seiji Kishi, Taichi Murakami, Tatsuya Tominaga, Kojiro Nagai, Hideharu Abe, Toshio Doi

**Affiliations:** Department of Nephrology, Institute of Biomedical Sciences, Tokushima University Graduate School, Tokushima, Japan; University of Louisville, UNITED STATES

## Abstract

Diabetic nephropathy (DN) is among the most serious complications of diabetes mellitus, and often leads to end-stage renal disease ultimately requiring dialysis or renal transplantation. The loss of podocytes has been reported to have a role in the onset and progression of DN. Here, we addressed the activation mechanism of Smad3 signaling in podocytes. Expression of RII and activation of Smad3 were induced by AGE exposure (P<0.05). Reduction of the activation of RII-Smad3 signaling ameliorated podocyte injuries in Smad3-knockout diabetic mice. The bone morphogenetic protein 4 (BMP4) significantly regulated activation of RII-Smad3 signalings (P<0.05). Moreover, the epithelium-specific transcription factor, Elf3was induced by AGE stimulation and, subsequently, upregulated RII expression in cultured podocytes. Induction of Elf3 and activation of RII-Smad3 signaling, leading to a decrease in WT1 expression, were observed in podocytes in diabetic human kidneys. Moreover, AGE treatment induced the secretion of Elf3-containing exosomes from cultured podocytes, which was dependent on the activation of the TGF-β-Smad3 signaling pathway. In addition, exosomal Elf3 protein in urine could be measured only in urinary exosomes from patients with DN. The appearance of urinary exosomal Elf3 protein in patients with DN suggested the existence of irreversible injuries in podocytes. The rate of decline in the estimated Glomerular Filtration Rate (eGFR) after measurement of urinary exosomal Elf3 protein levels in patients with DN (R^2^ = 0.7259) might be useful as an early non-invasive marker for podocyte injuries in DN.

## Introduction

Diabetic nephropathy (DN) is a morbid complication associated with diabetes mellitus and is the leading cause of end-stage renal disease (ESRD) [1]. Recent investigations have revealed that injuries in podocytes play a critical role in the development of many glomerular diseases, including DN [2–4]. In addition, the involvement of podocyte loss in the progression of DN has been reported [3–5]. Podocytes are highly specialized epithelial cells located on the surface of the glomeruli capillaries and normally prevent leakage of proteins into urine [2, 6]. Since these cells are unable to divide, their injury and malfunction leads to proteinuria, accumulation of extracellular matrix (ECM) components, and eventually results in irreversible glomerulosclerosis.

Various mechanisms including hyperglycemia, advanced glycation end-products (AGEs), protein kinase C, and activation of cytokines have been proposed as the cause of DN. Hyperglycemia increases the expression of transforming growth factor-β (TGF-β) in glomeruli and of matrix proteins specifically stimulated by this cytokine. TGF-β may contribute to the cellular hypertrophy and enhanced collagen synthesis observed in DN. TGF-β also plays an important role in the AGE response of the glomeruli [7], and transgenic mice overexpressing TGF-β develop severe glomerulosclerosis [8]. Thus, TGF-β is assumed to be a central mediator of the sclerosing process in DN. Based on amino acid sequence homology and functional properties, the TGF-β superfamily receptors are divided into two branches: type I and type II receptors. First, TGF-β binds to the cell surface type II receptor (RII), forming a heterodimer capable of recruiting and activating the type I receptor (RI). In the absence of RII, TGF-β has no affinity for RI. This receptor/ligand complex phosphorylates the Smad3 protein, which then enters the nucleus and regulates the transcription of a subset of genes related to cell proliferation. However, upstream signaling transduction pathways involved in the enhancement of TGF-β signaling in podocytes remain unknown. Transcriptional upregulation of RII is one of the mechanisms leading to TGF-β signaling activation. Some reports demonstrated that the transcription factor Elf3 interacts with purine-rich sequences in the RII promoter region [9]. However, the role of Elf3 in glomeruli has not yet been determined.

Screening for DN must be initiated at the time of type 2 diabetes diagnosis in patients, since ~7% of those have already microalbuminuria at that time [10]. DN is a progressive kidney disease caused by alterations in kidney architecture and function and constitutes one of the leading causes of ESRD. Although microalbuminuria is a widely used indicator for DN, its diagnostic accuracy is limited by the fact that structural damages might precede albumin excretion [11]. Therefore, it is important to find a novel diagnostic marker specific for the detection of podocyte injuries in DN, along with the elucidation of the molecular mechanisms leading to those injuries. Exosomes are 40–100 nm membrane vesicles secreted into the extracellular space by numerous cell types. These structures can be isolated from body fluids including urine and plasma [12]. Although exosomes were first described 30 years ago [13], these extracellular vesicles were largely ignored, forgotten, or dismissed as a means of cellular waste disposal. But in recent years, urinary exosomes have become an appealing source for biomarkers discovery as they contain molecular constituents of their cell of origin, including proteins and genetic material, and they can be isolated in a non-invasive manner [12]. In this study, we evaluated the significance of urinary exosomal Elf3 as a non-invasive diagnostic and predictive biomarker in patients with DN.

## Materials and methods

### Animals

Animals were housed under specific pathogen-free conditions at the animal facility of Tokushima University. All animal experiments were performed in accordance with the institutional guidelines, and the Review Board of Tokushima University granted ethical permission for this study. Six-week-old male C57BL/6 (WT), *BKS/Cg-m*^+/+^ Lepr db (db/db) and *BKS/Cg-m*^*+/+*^ Lepr db (db/+) mice were obtained from Charles River Japan (Tokyo, Japan) as previously described [14]. In the present study, we assessed Smad3+/- mice for a comparatively long period to examine the effects of Smad3 expression and phosphorylation and to evaluate the involvement of Smad3 in DN. Heterozygous *Smad3-* knockout mice were kindly provided by Dr. Yasue, University of Tokushima. We attempted to generate *Smad3*-null mice using pairs of *Smad3*^*+/-*^ mice, but progeny was rarely obtained, and pups were fragile and could not survive for a long period (5 weeks at the most). Therefore, *BKS/Cg-m*^*+/+*^ Lepr db (db/db) x *Smad3*^*+/-*^ mice were developed using pairs of Lepr db^+/-^ x *Smad3*^*+/-*^. *Smad3*^*+/-*^;db/+ mice were generated by crossing *Smad3*^*+/-*^ and db/+ mice. Moreover, *Smad3*^*+/-*^;db/db were generated by crossing *Smad3*^*+/-*^;db/+ and db/+ mice as previously described [14]. Blood glucose concentrations were measured from the tail vein (glucose assay kit; Abbott). The diabetic phenotype was confirmed 4 weeks after birth by blood glucose >300 mg/dl.

### Patients

Fifty patients with overt or heavy proteinuria and five healthy subjects were enrolled in this study. The recruitment took place at the Department of Nephrology of the Tokushima University Hospital, from April 2001 until March 2015. DN (n = 25) and minimal change nephrotic syndrome (MCNS;n = 25) were proven by renal biopsy. Type 2 diabetes mellitus was diagnosed according to the Japan Diabetes Society criteria. All the procedures were performed in accordance with the guidelines of the Helsinki Declaration on Human Experimentation and the Ethical Guidelines on Clinical Research published by the Japanese Health, Labor and Welfare Ministry. This study was approved by the Ethics Committee of Tokushima University, and informed written consent was obtained from all patients and healthy subjects as previously described [12]. The average age at biopsy was 48.2 ± 15.9 years, and the mean follow-up time after renal biopsy was 51.6 months (range: 7–98 months).

### Histological studies

Histopathological studies were performed on human tissues. Kidney specimens (n = 50) were obtained from renal biopsies. Kidney tissue blocks for light microscopy examination were fixed with Dubosq-Brazil solution and embedded in paraffin. Portions of kidney slices were mounted in Tissue-Tek OCT compound (Sakura Finetechnical, Tokyo, Japan) and snap-frozen. Multiple sections were prepared and stained with periodic acid silver methenamine (PAM) and periodic acid-Schiff’s reagent (PAS). For immunofluorescence, cryopreserved kidney sections were treated as previously described [15, 16]. Sections were incubated with the anti-podocin, anti-RII, anti-WT1 (Santa Cruz, CA), anti-pSmad3 (Cell Signaling Technology), and anti-Elf3 (Abcam) antibodies followed by incubation with the appropriate fluorescent secondary antibodies.

### Cell culture

Conditionally immortalized murine podocytes were purchased from Cell Lines Service (CLS, Eppelheim, Germany) and were propagated under permissive condition at 33°C, as previously described [17]. Podocytes were grown in RPMI 1640 medium (Gibco, Invitrogen, Carlsbad, CA, USA) containing 4 mM glutamine, 5% exosome-free fetal bovine serum (FBS), and 100 units/ml penicillin/streptomycin. Podocytes between passage 5 and 10 were cultured on plates. Cells were passaged routinely at 80% confluence by using Accutase (Millipore, Billerica, MA), as previously described [12]. To induce differentiation, cells were switched to 37°C (nonpermissive condition). To examine the expression and activation of the RII-Smad3 signaling pathway in cultured podocytes in a diabetic condition, podocytes were treated with AGE. After a 48-h exposure to AGE or BSA (5μg/ml) at 37°C, conditioned media were collected and proteins were isolated from podocytes. Equal amounts of cell lysates or conditioned media were subjected to western blotting. Treatment with a BMP4-neutralizing antibody or control IgG antibody (R&D Systems) was performed. AGE-BSA was prepared by the method described previously [18].

### Western blotting

Cell lysates were suspended in RIPA buffer (50mM Tris, pH 7.5, 150 mM NaCl, 1% Nonidet P-40, 0.25% SDS, 1mM Na_3_VO_4_, 2mM EDTA, 1mM phenylmethylsulfonyl fluoride, 10 mg/ml aprotinin) and incubated for 1 h at 4°C. After electrophoresis, proteins were transferred onto a nitrocellulose membrane (Hybond-ECL, GE Healthcare). Membranes were incubated with anti-Smad3 (Abcam), pSmad3 (Cell Signaling Technology), α-tubulin, Elf3, RII, and WT1 (Santa Cruz Biotechnology) antibodies, followed by incubation with horseradish peroxidase-conjugated secondary antibodies (GE Healthcare). The immunoreactive bands were visualized using an ECL Western blotting detection system (GE Healthcare).

### Plasmid and transfection

The full-length cDNA of Elf3 was obtained using gene-specific primers for reverse transcription-PCR (RT-PCR) and was cloned into pcDNA3-GFP. The constructed plasmid was verified by DNA sequencing. Cells cultured at 33 °C were electroporated (1x10^6^ cells, 25 ms, 1300 V) with 10 μg of the Elf3-expressing plasmid, using the Neon Transfection System (Invitrogen). After transfection, cells were transferred to 37 °C, allowed to differentiate for 48 h, and used in subsequent experiments. Using GFP as a reporter gene, the transfection efficiency of a population of podocytes was determined as the percentage of cells expressing GFP in the entire population.

### Small-interfering RNA

Small-interfering RNAs (siRNAs) for Elf3 or control scrambled siRNA were purchased from Dharmacon. Cells were electroporated (1x10^6^ cells, 25 ms, 1300 V) with 3 μg of siRNA, using the Neon Transfection System. Cells were then resuspended in complete RPMI medium, incubated for 48 h, and used in subsequent experiments.

### Urine preparation and isolation of exosome from urine and conditioned media

Urine samples (50–150 ml) were collected from patients (before biopsy) and healthy subjects in sterile containers and centrifuged at 300 x*g*, using a fixed-angle rotor for 10 min at room temperature (within 1 hour of collection) to remove any particulate matter, including cells and cell debris as previously described [12]. The clarified urine was stored at -80°C before further analysis. In addition, upon reaching near confluence, conditioned media of cultured podocytes were collected, and differential centrifugation was performed. Conditioned media were centrifuged at 300 x*g* for 10 min, 2000 x*g* for 20 min, and 10,000 x*g* for 40 min. Cleared conditioned media were then concentrated using a 3-kDa MWCO concentrator (Millipore) to <5ml. The supernatants from urine and conditioned media were ultracentrifuged for 1 h at 70,000 x*g* using an angled rotor (RP-65; Hitachi Koki Co, Ltd.). Pellets were resuspended in 5ml of 0.25M sucrose, 20mM HEPES-NaOH (pH 7.2) and poured carefully on top of 30ml sucrose density gradients [2–0.25M sucrose, 20mM HEPES-NaOH (pH 7.2)] in 40 PA centrifuge tube (329607A; Hitachi Koki Co, Ltd.). Samples were ultracentrifuged for 20h at 100,000 x*g* using a swing rotor (RPS-28SA, Hitachi Koki Co, Ltd.) and fractionated to 12 2 ml samples in 5 PA centrifuge tubes (332245A; Hitachi Koki Co, Ltd.) by collecting from the bottom of the tube. Fractions were diluted with 3 ml PBS and ultracentrifuged for 1 h at 200,000 x*g* using a swing rotor (RPS-50-2; Hitachi Koki Co, Ltd.). Pellets were resuspended in 50μl PBS. The recovery efficiency and purity were analyzed by silver staining and western blotting as previously described [12].

### Statistical analysis

Data are expressed as the mean ± standard error (S.E.). Statistical analysis for comparison was performed using one-way analysis of variance followed by the Tukey’s multiple comparisons test. P<0.05 was considered to indicate a statistically significant difference. Creatinine adjustment is widely used for the estimation of 24-h urinary creatinine excretion from spot urine samples. The distribution of the exosomal Elf3 expression levels was obtained based on the values of corrected Elf3, which were divided by the urinary creatinine concentration. For the analysis of a correlation between exosomal Elf3 expression levels and the predictive power for the prognosis of renal function, a correlation curve was obtained by plotting the quantification results of the corrected Elf3 value.

## Results

### Reduction of expression and activity of Smad3 improves DN in mice

**Fig 1:**
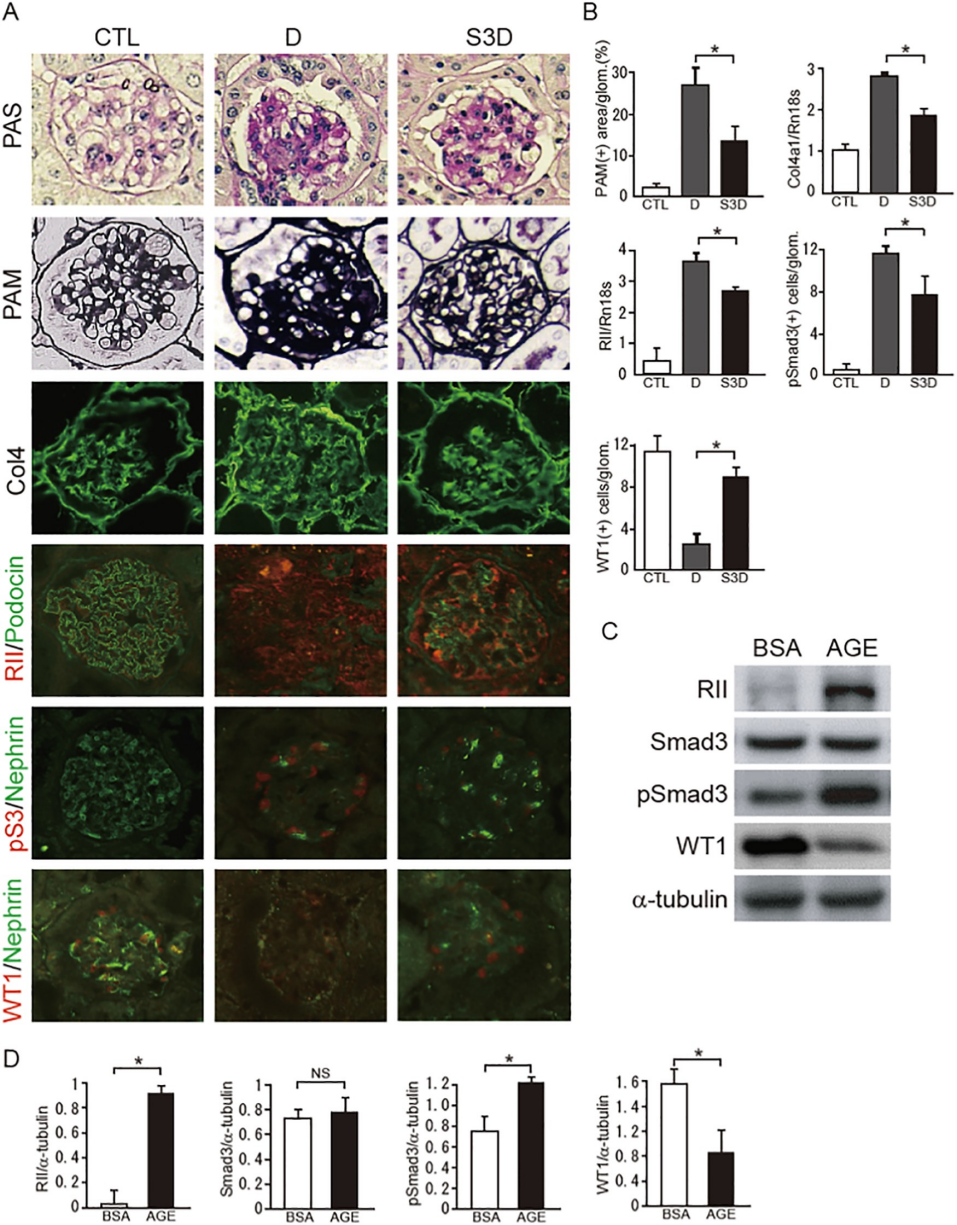
Effect of Smad3 deletion on phenotypic changes on podocytes in diabetic nephropathy. (A) Representative photomicrographs of periodic acid-Schiff methenamine (PAM), periodic acid-Schiff staining (PAS), and immunohistochemical staining are shown (original magnification x400). The left panels are data from normal control (CTL) mice and the middle panels are from db/db mice (D), and the right panels are from *Smad3*-knockout diabetic mice (Smad3+/-;db/db mice) (S3D). (B) The mesangial sclerotic fraction was determined in the three types of mice as the percentage of mesangial matrix area per total glomerular surface area. All glomeruli were analyzed in each sample. The levels of Col4 (Col4a1) and RII mRNA in the glomeruli were analyzed by qPCR and normalized to the expression of Rn18s. Regarding pSmad3 and WT1, the number of phosphorylated Smad3- and WT1- positive nuclei were counted, and the mean values (per glomerulus) were calculated. The values are expressed as the mean ± S.E. *P<0.05. (C) Western blot of protein extracts prepared from cultured podocytes treated with AGE or BSA (5 μg/ml) for 24 h. Equal amounts of cell lysates were subjected to Western blotting. One of three independent experiments is shown. α-tubulin was used as loading control. (D) Optical densitometry of the bands in western blot. Values are expressed as the mean ± S.E. (NS, not significant, *, p<0.05, t-test).

In Smad3+/-;db/db mice, both PAS- or PAM-positive mesangial areas and type IV collagen expression levels were suppressed compared with those in db/db mice (Fig 1A and 1B). In diabetic mice, a decreased expression of podocyte-specific proteins, such as podocin [19] and nephrin [20] was observed as previously described (Fig 1A and 1B). Moreover, glomerular WT1 expression was significantly decreased in db/db mice compared with Smad3+/-;db/db mice, suggesting that activation of Smad3 signalings is involved in podocyte damage. RII is a type II receptor for TGF-family proteins and, specifically, phosphorylates Smad3. Furthermore, glomerular RII expression was decreased in Smad3+/-;db/db mice compared with that in db/db mice (Fig 1A and 1B). We were able to detect a significant induction of RII and activation of Smad3, and decreased expression of WT1 protein in AGE-treated podocytes (Fig 1C and 1D). As a result, phosphorylation of Smad3 was enhanced in AGE-treated podocytes. In contrast, BSA did not phosphorylate Smad3. Additionally, expression of WT1 protein was reduced in AGE-treated podocytes, suggesting that activation of RII-Smad3 signaling causes podocyte injuries. These data suggest that repression of Smad3 expression in diabetic glomeruli attenuates DN progression.

### Elf3 is induced by BMP4 in cultured podocytes

**Fig 2:**
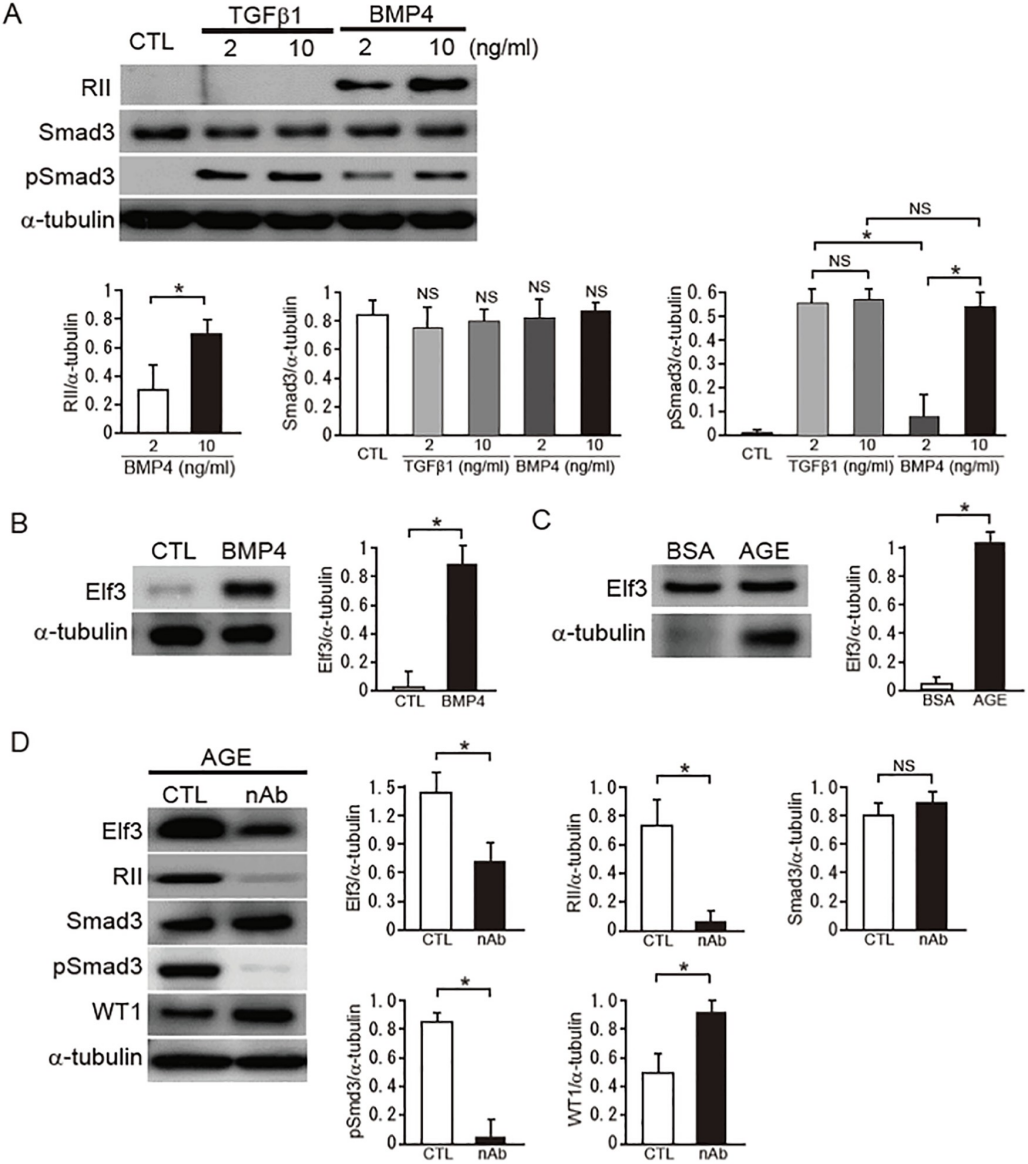
Induction of RII in podocytes via BMP4 in a diabetic condition. (A) Effects of TGFβ1 and BMP4 on the expression of RII and activation of Smad3 in cultured podocytes. (B) Effects of BMP4 on the expression of Elf3 in cultured podocytes. (C) Expression of Elf3 in cultured podocytes treated with AGE or BSA (5 μg/ml) for 24 h. (D) Podocytes were treated with a neutralizing antibodies for BMP4 (nAb) (10 μg/ml) or control normal IgG (CTL) for 24 h after a 24-h exposure to AGEs (5 μg/ml). Equal amounts of cell lysates were subjected to western blotting. One of three independent experiments is shown. α-tubulin was used as loading control. Quantitation of these proteins expression was assessed by quantitative PCR analysis and normalized to the expression of α-tubulin.

We previously demonstrated that BMP4, as well as TGF-β1, is involved in the process of glomerulosclerosis (16). To further elucidate the mechanism of RII expression and function in a diabetic condition, we investigated the expression of RII in cultured podocytes treated with TGF-β1 or BMP4. We were able to detect a significant induction of RII protein expression upon BMP4 stimulation (Fig 2A). In contrast, activation of Smad3 was clearly observed in TGFβ1-treated podocytes, because RII is a type II receptor for TGF-β1 and specifically phosphorylates Smad3 (Fig 2A). Based on these results, we examined the expression levels of Elf3 in BMP4-treated podocytes. The induction of Elf3 by BMP4 was eminent in consistent with the increase in RII protein levels induced by BMP4 (Fig 2B). In addition, AGE treatment also upregulated the expression of Elf3 protein (Fig 2C). To confirm the role of BMP4 in AGE exposure in cultured podocytes, we performed an inhibition assay using a BMP4-specific neutralizing antibody. Antibody-mediated neutralization of BMP4 markedly reduced the expression of Elf3 and RII, and the activation of Smad3, resulting in the protection from the decrease in WT1 expression (Fig 2D). Collectively, Elf3 is significantly induced by BMP4 and is involved in podocyte damage through the activation of RII-Smad3 signaling in a diabetic condition.

### Elf3 plays a key role in regulating podocyte damage in DN

**Fig 3:**
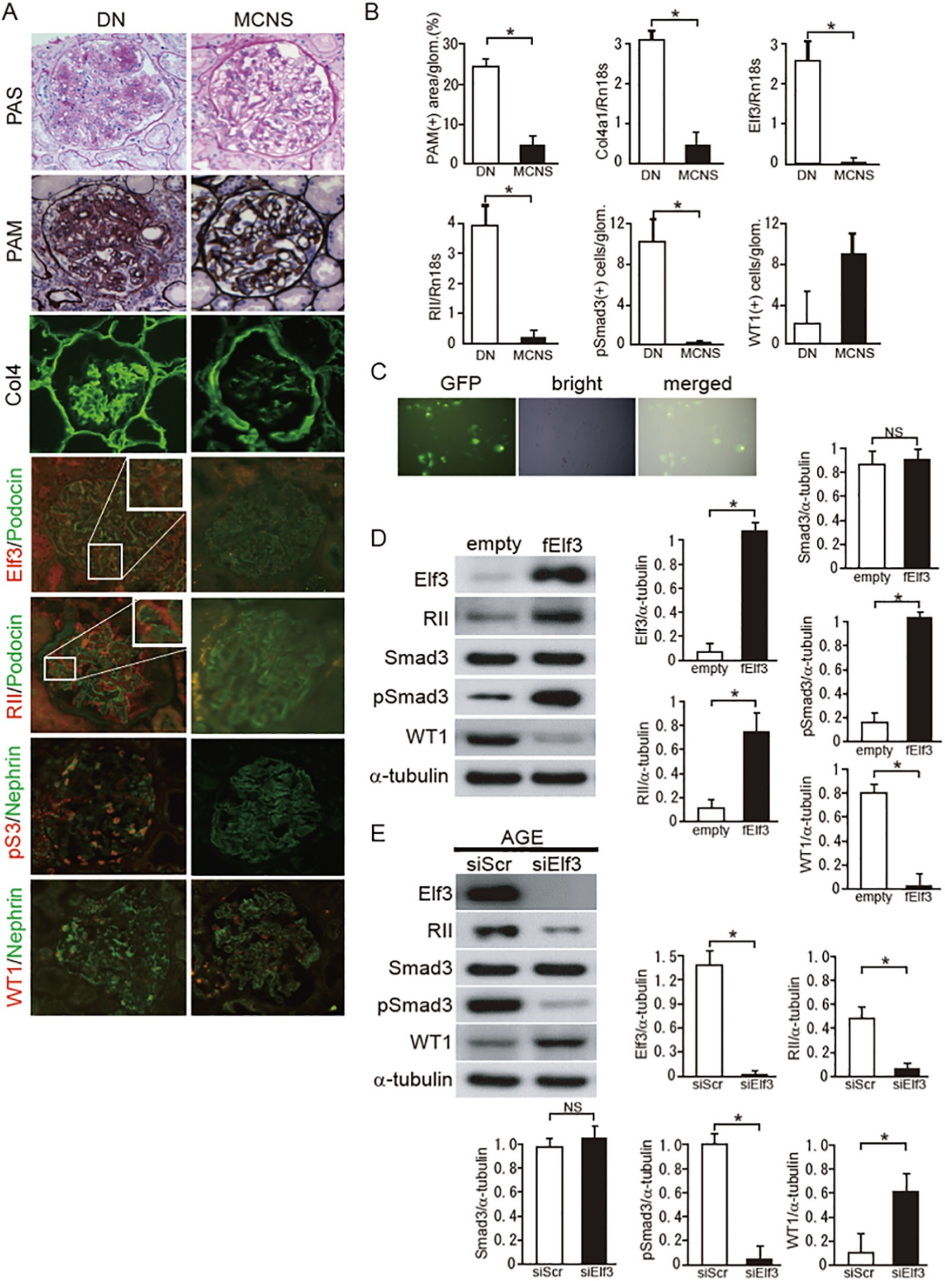
Glomerular expression of Elf3 and Smad3 signaling-related proteins in patients with DN and with MCNS. (A) Representative photomicrographs of periodic acid-Schiff methenamine (PASM), periodic acid-Schiff staining (PAS), and immunohistochemical staining of Col4, Elf3, RII, pSmad3 (pS3) and WT1 are shown (original magnification: x400). The left panels contain data from patients with DN, and the right panels show data from patients with MCNS. (B) The mesangial sclerotic fraction in patients with DN and with MCNS was determined as the percentage of mesangial matrix area per total glomerular surface area. Regarding pSmad3 and WT1, the number of phosphorylated Smad3- and WT1- positive nuclei was counted, and the mean values (per glomerulus) were calculated. Values are expressed as the mean ± S.E. *P<0.05. (C) Representative immunofluorescence and microscopic images for Elf3 (green: GFP) 48 h post-transfection. (D) Effects of overexpression of full-length Elf3 (fELF3) vector or empty vector in cultured podocytes. (E) Control siRNA (scrambled) or siRNA specific for Elf3 were transfected into podocytes. Ablation of Elf3 expression following siRNA knockdown in cultured podocytes was confirmed by immunocytochemistry. Equal amounts of cell lysates were subjected to western blotting. One of three independent experiments is shown. α-tubulin was used as a loading control. Quantitation of these proteins expression was assessed by quantitative PCR analysis, and normalized to the expression of α-tubulin.

We investigated the association between glomerular expression of Elf3 and podocyte injuries in patients with DN and patients with MCNS. We first observed that the number of WT1-positive podocytes was significantly reduced in patients with DN compared with the MCNS group (Fig 3A), as previously reported [21]. Glomerular immunoreactivity for Elf3 was correlated with the severity of sclerotic lesions in diabetic renal glomeruli, and the immunoreactive signal was nearly absent in glomeruli of MCNS (Fig 3A). As expected, the increase in RII expression and the activation of Smad3, as well as the decrease in WT1 expression, were observed in patients with DN (Fig 3A and 3B). These histological observations suggest that the induction of Elf3 is involved in podocyte injuries in DN. To further elucidate the relationship between Elf3 and Smad3 signaling pathway in podocytes, Elf3 was overexpressed in podocytes by transient transfection of an Elf3-expression plasmid. Podocytes were successfully transfected with the full-length mouse Elf3 cDNA together with GFP as indicated by the formation of green fluorescent clusters (Fig 3C). The forced expression of Elf3 induced the activation of RII-Smad3 signaling and subsequently decreased the expression of WT1. These results suggest that Elf3 significantly regulated WT1 expression via activation of Smad3 in podocytes independently of the presence of a diabetic state (Fig 3D). Next we investigated whether Elf3 has these regulatory effects on Smad3 activation and podocyte injuries upon AGE stimulation. Knockdown of Elf3 by RNA interference suppressed RII-Smad3 signals and restored WT1 expression in podocytes stimulated by AGE (Fig 3E). These results indicate that Elf3 plays an important role in podocyte injuries through the activation of RII-Smad3 signaling in a diabetic condition.

### Cultured podocyte-derived Elf3 protein in exosomes in a diabetic condition

**Fig 4:**
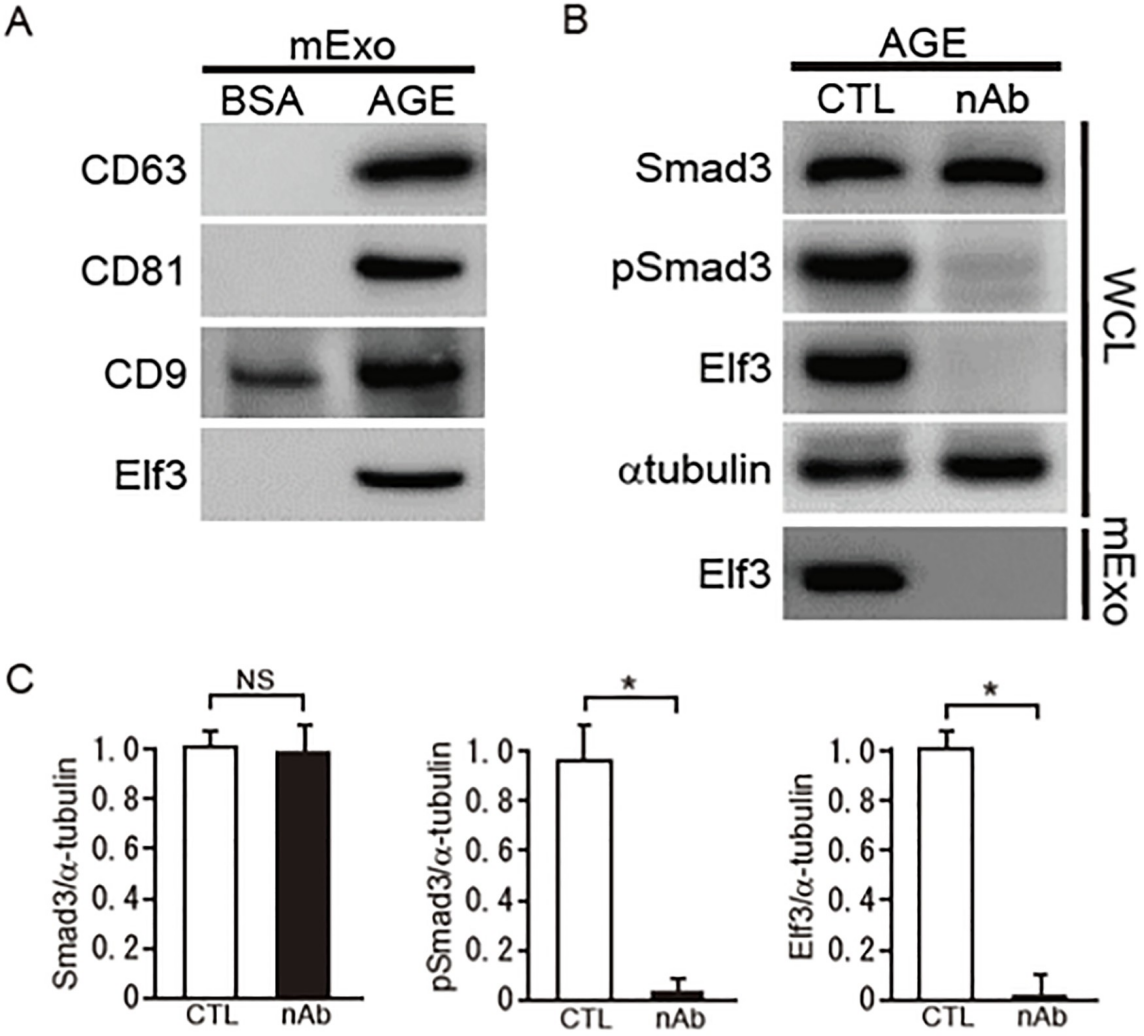
Elf3 protein in exosome in media from cultured podocytes exposured to AGE. (A) Expression of Elf3 protein in mouse podocytes cultured in exosome-free medium under exposure to BSA or AGE (5 μg/ml) for 48 h was monitored by western blotting. Western blot analysis for CD63, CD81, and CD9 as exosome markers for exosomes obtained from conditioned media (mExo) from cultured podocytes. (B) Podocytes were treated with neutralizing antibodies for BMP4 (nAb: 10 μg/ml) or control normal IgG (CTL) for 24 h after a 24-h exposure to AGE (5 μg/ml). Equal amounts of whole cell lysates (WCL) or exosomal proteins obtained from conditioned media (mExo) were subjected to western blotting. One of three independent experiments is shown. α-tubulin was used as a loading control. (C) Optical densitometry of these proteins in western blot was shown. The values are expressed as the mean ± S.E. (NS, not significant, *, p<0.05, t test).

Recently, it has been reported that podocytes release exosomes into urine as a footprint of cellular functional processes [22]. In addition, Elf3 is already known to be secreted in exosomes and has been registered in the database ExoCarta (http://exocarta.ludwig.edu.au/, April 2011 version), which is specially dedicated to exosomes from various species and their components [22]. To elucidate the mechanisms of exosome secretion from podocytes, we isolated exosomes from conditioned media and examined the expression of Elf3 in exosomes secreted from cultured podocytes subjected to AGE stimulation. We first detected tetraspanins classically used as exosome markers (CD9, CD63, and CD81) (Fig 4A). Expression of Elf3 in exosomes from podocytes treated with AGEs was also observed. In contrast, BSA stimulation used as control did not induce the expression of Elf3 protein in exosomes (Fig 4A). To confirm whether the secretion of Elf3 in exosomes is regulated by the activation of the TGF-β-Smad3 signaling pathway, an inhibition assay using the TGF-β-neutralizing antibody was performed. Podocytes treated with the BMP4-neutralizing antibody showed a significant reduction of Elf3 in exosomes obtained from conditioned media (Fig 4B and 4C). These findings suggest that BMP4 stimulates the secretion of Elf3 protein in exosomes as well as the induction of Elf3 protein in cultured podocytes in a diabetic condition.

### Urinary exosomal Elf3 protein is associated with podocyte injuries in patients with DN

**Fig 5:**
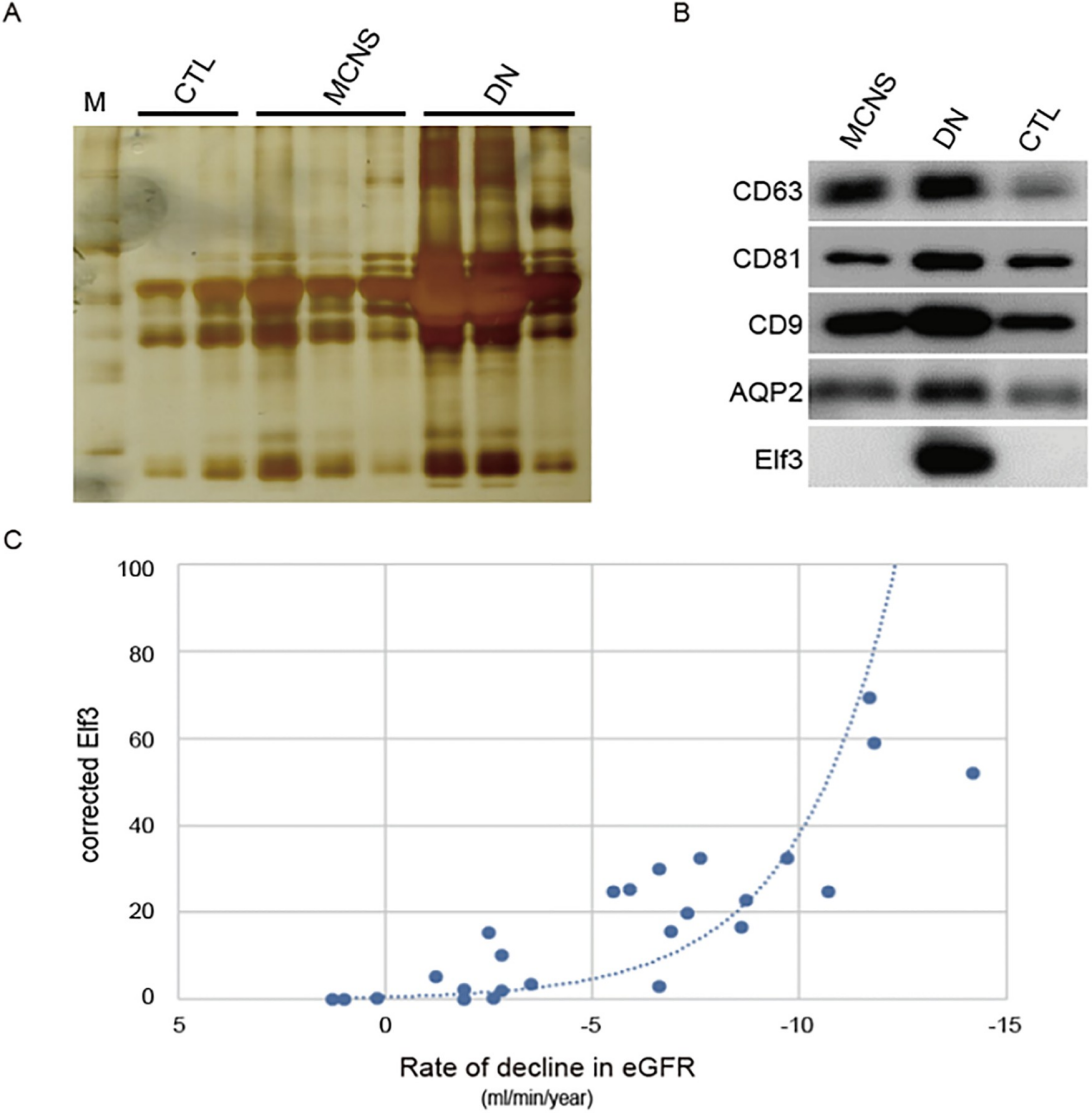
Expression levels of Elf3 and changes in eGFR after measurement of Elf3. (A) Urinary exosomes obtained from patients with MCNS and DN, and healthy subjects (CTL) were assayed by SDS/PAGE and visualized by silver staining. The molecular weight marker was indicated on the left. (B) Western blotting of urinary exosomes in pooled urine samples collected from patients with DN (n = 25) and MCNS (n = 25), and healthy subjects (CTL) (n = 5) using antibodies specific for CD63, CD81, and CD9. (C) Rate of change in eGFR after the measurement of urinary exosomal Elf3 protein levels in the patients with DN. R^2^ = 0.7259.

Next, we investigated whether exosomal Elf3 protein is secreted from patients with proteinuria. First, to determine whole exosomal proteins levels in the urine, exosomes obtained from patients with DN, patients with MCNS, and healthy subjects were analyzed by silver staining (Fig 5A). The loading volume for immunoblotting was normalized to the urinary creatinine content. The amount of exosomes obtained from patients with DN was higher than that obtained from patients with MCNS or healthy subjects. Next, we examined whether aquaporin-2 (AQP2) is expressed in the exosomes from the three groups, as excretion of AQP2 in urinary exosomes has been reported in healthy subjects who had no evidence of recent kidney or urinary tract disease [23]. While AQP2, as well as tetraspanins, could be detected in all three groups, by western blotting, excretion of Elf3 in urinary exosomes was restricted to the urine obtained from patients with DN (Fig 5B). This is compatible with the fact that glomerular expression of Elf3 protein could not be observed in patients with MCNS. Based on these results, the clinical linkage between the elevated value of urinary exosomal Elf3 protein and the rapid decline in kidney function was examined in patients with DN. During the follow-up period, 20.0% (5/25) of the patients with DN developed ESRD (Fig 5C). To visualize the relationship between the corrected Elf3 value and the rate of decline in the estimated Glomerular Filtration Rate (eGFR) in each patient with DN and its trend, a scatterplot is shown in Fig 5C. The correlation curve with a high coefficient of determination (R^2^ = 0.7259) was obtained, suggesting that urinary exosomal Elf3 can be a molecular marker for podocyte injuries and can predict the decline in eGFR in the coming years.

## Discussion

According to the World Health Organization, the number of people with diabetes mellitus has risen from 108 million in 1980 to 422 million in 2014 [24]. Moreover, DN is associated with an increased risk of adverse cardiovascular outcomes, morbidity, and mortality. Podocyte structural changes have been suggested to be involved in the pathogenesis of albuminuria in diabetes. Studies in rats with reduced nephron numbers and consequent glomerular hypertrophy have demonstrated that podocytes suffer progressive injuries when forced to cover a larger surface area [25]. Thus far, some reports suggest that activation of Smad3 signaling is an important event for podocyte injury [26, 27]. However, the precise role of Smad3 in podocytes, including transcriptional regulatory mechanisms for the phosphorylation of Smad3 are unclear. Because normal glomeruli exhibit steady-state Smad3 expression, we conducted a study using *Smad3*+/-;db/db mice. We observed that db/db mice showed severe glomerulosclerosis and the increase in Col4 expression and Smad3 activation. These changes were significantly attenuated in *Smad3*+/-;db/db mice. Moreover, the reduction in the number of WT1-positive podocytes is alleviated in *Smad3*+/-;db/db mice compared with db/db mice, suggesting that Smad3 activation is involved in podocyte damage in diabetes mellitus. TGF-β directly binds to TGF-β RII, which leads to the phosphorylation of TGF-β RI. This phosphorylation activates the RI protein kinase, that then, phosphorylates Smad3 [28]. In *Smad3*+/-;db/db mice, induction of RII expression was also significantly attenuated compared with db/db mice. Taken together, these results suggest that induction of RII expression is an important event in the development of DN.

We recently reported on the interplay between Smad1 and Smad3 under AGE stimulation in the progression of DN [14]. In addition, we previously demonstrated that BMP4 plays a critical role in the initiation of DN using conditional BMP4 transgenic mice [29]. To clarify the molecular mechanisms underlying the RII-Smad3 signaling pathway activation in podocytes, we examined the effects of TGFβ1 and BMP4 on this pathway. Although Smad3 protein expression levels were not changed, BMP4 significantly induced the expression of RII compared with the induction with TGF-β1. On the other hand, TGFβ1 activated Smad3 in cultured podocytes as expected. It was previously reported that RII is transcriptionally regulated by Elf3 in some cancer cell lines [30, 31]. Therefore, we investigated whether Elf3 is involved in the regulation of RII expression in cultured podocytes. Elf3 was induced by BMP4 and regulated the RII-Smad3 signaling pathway in podocytes treated with AGEs, suggesting that Elf3 plays an important role in the activation of Smad3 via the induction of RII and subsequent podocyte damage.

Ets transcription factors are expressed exclusively in tissues with a high content of epithelial cells including skin, small intestine, colon, and kidneys. Podocytes, also called glomerular visceral epithelial cells, are terminally differentiated, highly specialized cells with a unique location, architecture, and relevance. Among Ets transcription factors, the epithelium-specific transcription factor Elf3 has been reported to modulate TGF-β signaling by regulating the transcription of RII in various cancer cells [30, 31]. In this study we first demonstrated that Elf3 is induced in diabetic glomeruli. These results suggest that Elf3 expression is induced by direct stress, such as high glucose and/or AGEs in podocytes. Therefore, ectopic expression of Elf3 in podocytes may be involved in the induction of abnormal signaling, including RII-Smad3 axis, leading to glomerulosclerosis in diabetes mellitus.

Since it is difficult to prevent the progression of nephropathy in overt nephropathy, treatment at the early phase is recommended. Podocyte damage or loss is a critical symptom presenting clinically with proteinuria and subsequent glomerulosclerosis. Therefore, it has been deemed necessary to find novel diagnostic molecular markers specific for the detection of podocyte injuries in the initial phase of DN [7, 13], along with the elucidation of the molecular mechanisms of the activation of TGF-β signaling in podocytes. In this study, we characterized the association between urinary exosomal Elf3 protein and kidney function in patients with DN. Elf3 is already registered as an exosomal protein in ExoCarta (http://exocarta.ludwig.edu.au/, April 2011 version), a database specially dedicated to exosomes from various species and their components [10]. As far as we know, we are the first ones identifying exosomal Elf3 protein in urine. Urinary exosomal Elf3 protein levels could discriminate DN from MCNS, despite the same level of proteinuria. From these facts, it is speculated that induction of Elf3 in podocytes may lead to critical phenotypic alterations coupled with irreversible damages. Because Elf3 is a member of the Ets family of transcription factors, whose expression is restricted to epithelial cells, and known to be involved in the regulation of epithelium-specific gene products [24, 25], Elf3 may play an important role in the process of phenotypic alterations of podocyte induced by the activation of TGF-β signals. Thus, this study proposes that quantification of Elf3 levels in urinary exosomes may be useful for evaluating podocyte injuries due to the activation of TGF-β signaling and that it may serve as a novel predictive marker of podocyte loss.
